# Colorants and Antioxidants Deriving from Methylglyoxal and Heterocyclic Maillard Reaction Intermediates

**DOI:** 10.3390/antiox12091788

**Published:** 2023-09-21

**Authors:** Leon Valentin Bork, Maximilian Baumann, Tobias Stobernack, Sascha Rohn, Clemens Kanzler

**Affiliations:** 1 Department of Food Chemistry and Analysis, Institute of Food Technology and Food Chemistry, Technische Universität Berlin, Gustav-Meyer-Allee 25, 13355 Berlin, Germany; m.baumann@tu-berlin.de (M.B.); rohn@tu-berlin.de (S.R.); 2 Department of Chemical and Product Safety, German Federal Institute for Risk Assessment, Max-Dohrn-Str. 8–10, 10589 Berlin, Germany; tobias.stobernack@bfr.bund.de

**Keywords:** Maillard reaction, non-enzymatic browning, methylglyoxal, norfuraneol, furfural, α-dicarbonyl compounds, pre-melanoidins, food browning, antioxidant colorants

## Abstract

The Maillard reaction is well known for producing antioxidant compounds alongside colored substances. Low-molecular-weight antioxidant intermediates such as maltol (MAL) or norfuraneol (NF) are well described, but it is still unclear which of these Maillard intermediates are the precursors of antioxidant and colored melanoidins—the so-called late stage Maillard reaction products. This study aimed to provide novel insights into the correlation between browning potential and antioxidant properties of reaction products formed during the heat treatment of prominent Maillard reaction intermediates. It was achieved by the incubation of binary reaction systems composed of methylglyoxal (MGO) or NF in combination with furfural (FF), MAL, and pyrrole-2-carbaldehyde (PA) at pH 5 and 130 °C for up to 120 min. Overall, it could be shown that the formation of colored products in the binary NF reaction systems was more efficient compared to those of MGO. This was reflected in an increased browning intensity of up to 400% and a lower conversion rate of NF compared to MGO. The colorants formed by NF and FF or PA (~0.34 kDa and 10–100 kDa) were also found to exhibit higher molecular weights compared to the analogue products formed in the MGO incubations (<0.34 kDa and 10–100 kDa). The incorporation of NF into these heterogenous products with FF and PA resulted in the preservation of the initial antioxidant properties of NF (*p* < 0.05), whereas no antioxidant products were formed after the incubation of MGO.

## 1. Introduction

Carbohydrate chemistry has a long and fruitful history in Germany with well-known scientists such as Emil Fischer, Franz Ledl, Thomas Severin, Erwin Schleicher, and Werner Baltes, who have contributed significantly to the understanding of the properties, stability, and reactivity of carbohydrates. The focus of their research was non-enzymatic browning reactions, especially the Maillard reaction of reducing carbohydrates and amino compounds. This complex reaction cascade is ubiquitous in nature and therefore, highly relevant in physiological systems, but even more prominent for processed food, as there, the reaction is significantly forced by thermal treatments. An important outcome of the Maillard reaction is the formation of low-molecular-weight volatile compounds, whose chemical structures and formation mechanisms are well described [1,2]. Among these, reductone ethers, such as maltol (MAL) and their 1,2-dicarbonyl precursors (‘reductones’), have been characterized as process-borne antioxidant compounds [3]. Subsequent reactions of such intermediates, most prominently 1,2-dicarbonyls [4,5] and active methylenes [6,7], with nitrogen-containing compounds are proposed to yield the colored and redox-active end-products of the Maillard reaction, known as melanoidins [8]. Their daily intake through the human diet is estimated to be around 10 g on a basis of frequently consumed food such as coffee and bread [9]. The consumption of melanoidins was found to be beneficial for the digestive system due to their antidiabetic [10], prebiotic [11], and antioxidant effects [12,13]. However, genotoxic [14] and pro-oxidative [15] properties are also discussed quite controversially in the context of an overconsumption of processed food [16,17,18]. It was estimated that the daily consumed amount of melanoidins contributes to around 20% of the total antioxidant activity of a Mediterranean diet, usually regarded as very healthy [19], whereas the cumulative antioxidant capacity of the recommended daily intake of vitamin C and vitamin E amounts to around 10% [20]. In essence, Maillard reaction products represent an underestimated group of antioxidant food constituents that can significantly contribute to the stability of processed food.

To understand and eventually control the color [21,22], antioxidant [23,24], and physiological properties [12,14,25,26,27] of food melanoidins, it is crucial to know their exact chemical structure to derive structure–activity relationships. However, the necessary data, especially regarding the exact chemical structure of melanoidins, are not comprehensive, so far. Additionally, there are confounding findings regarding the correlation between color formation and the antioxidant properties of Maillard reaction products. On one hand, it was reported that the antioxidant activity of low-molecular-weight Maillard reaction products in coffee was higher compared to those of high-molecular-weight melanoidins [28,29], but the intense browning is more attributed to the high-molecular-weight products [28]. Researchers assumed that the incorporation of smaller compounds into macromolecular melanoidins accounted for the elevated antioxidant activity of melanoidins [29]. On the other hand, the positive correlation between color and antioxidant activity of Maillard reaction products is well established based on model experiments of sugars and amino compounds [30]. For example, high-molecular-weight melanoidins (>300 kDa) isolated after the incubation of glucose/asparagine or fructose/asparagine were characterized by a higher browning intensity and antioxidant activity compared to low-molecular-weight compounds (<50 kDa) [31]. Such findings demonstrate that the properties of these complex, heterogenous group of polymers are strongly dependent on the reaction conditions and most prominently, on the initial reactants of the reaction. The same principles apply to the chemical structure of melanoidins, because the physicochemical properties are defined by the structure.

To build an in-depth understanding of the characteristic properties of melanoidins, such as their antioxidant activity, color, and molecular weight, studies on the reactivity of selected key compounds have become a valuable approach in Maillard reaction research [32,33,34,35]. In contrast to complex reaction mixtures or real food systems, in which an unmanageable number of reactants undergoes consecutive and parallel reactions, forming heterogenous, complex statistical copolymers, model systems of selected intermediates enable a more systematic investigation of selected reaction pathways and their corresponding products.

**Figure 1:**
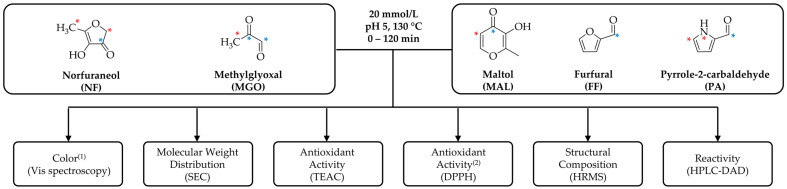
Experimental and analytical approach for the characterization of antioxidant colorants formed by key Maillard reaction intermediates. Norfuraneol (NF) or methylglyoxal (MGO) were incubated in binary, equimolar mixtures with maltol (MAL), furfural (FF), and pyrrole-2-carbaldehyde (PA) at pH 5 and 130 °C for 120 min. Nucleophilic (*) and electrophilic (*) centers which were hypothesized to enable the formation of heterogenous reaction products are highlighted. ^(1)^ Color analysis was performed for the aqueous solutions and for the corresponding ethyl acetate extracts. ^(2)^ Antioxidant activity of predominantly apolar reaction products was only analyzed for the ethyl acetate extracts.

The present study aimed to characterize the reactivity of different groups of key Maillard reaction intermediates, which can be described by their antioxidant properties and/or their browning potential (Figure 1). For this purpose, the browning precursors furfural (FF), pyrrole-2-carbaldehyde (PA), or the well-known antioxidant reductone MAL were incubated in binary model systems at 130 °C for up to 120 min in combination with the active methylene and reductone ether norfuraneol (NF) and the 1,2-dicarbonyl methylglyoxal (MGO). It was hypothesized that the increased reactivity and browning potential of NF [36,37] and MGO [38,39] results from their high density of nucleophilic (*) and electrophilic (*) centers (Figure 1). By incubating with different carbonyl compounds, this study is the first to investigate the question of whether antioxidant monomers preserve their properties after being incorporated into colored Maillard products and if colorants with even improved antioxidant properties compared to those of their precursors are formed throughout non-enzymatic browning reactions.

To characterize the different model incubations, reactivity was analyzed by the conversion of the reactants with high-performance liquid chromatography coupled to a diode array detector (HPLC-DAD) and their browning potential was determined by Vis spectroscopy. The colored reaction mixtures were additionally analyzed by size-exclusion chromatography (SEC), high-resolution mass spectrometry (HRMS), and the trolox equivalent antioxidant capacity assay (TEAC). Additionally, the contribution of apolar reaction products to the color and the lipophilic antioxidant activity (2,2-diphenyl-1-picrylhydrazyl (DPPH) radical scavenging) was determined after extraction in ethyl acetate (EE). The structural composition of redox active reaction products obtained by HRMS were used to propose a structure–activity relationship between the color, molecular weight, and antioxidant properties of melanoidins.

## 2. Materials and Methods

### 2.1. Chemicals

Acetonitrile, hydrochloric acid (37 wt.%), ethyl acetate, and methanol were purchased from VWR International GmbH (Darmstadt, Germany). 2,2′-Azinobis(3-ethylbenzothiazoline-6-suflonate) (ABTS), furfural (FF, furan-2-carbaldehyde), maltol (MAL, 3-hydroxy-2-methyl-pyran-2-one), methylglyoxal (MGO, 2-oxopropanal) (40 wt.%), norfuraneol (NF, 4-hydroxy-5-methyl-3-(2*H*)-furanone), potassium persulfate, and trolox (6-hydroxy-2,5,7,8-tetramethylchroman-2-carboxylic acid) were purchased from Sigma-Aldrich GmbH (Steinheim, Germany). Potassium dihydrogen phosphate and potassium hydrogen phosphate were purchased from Merck KGaA (Darmstadt, Germany). 2,2-Diphenyl-1-picrylhydrazyl radical was purchased from TCI International GmbH (Hamburg, Germany). Pyrrole-2-carbaldehyde (PA, 1*H*-pyrrole-2-carbaldehyde) was purchased from Acros Organics N.V. (Geel, Belgium). Sodium hydroxide pellets were purchased from Carl Roth GmbH + Co. KG (Karlsruhe, Germany). 

### 2.2. Incubation of Maillard Reaction Intermediates in Aqueous Solution

Equimolar (20 mmol/L) binary model systems were prepared for the combinations of NF (57.0 mg) and MGO (aqueous solution with 40 wt.%, 90.1 mg) as well as NF or MGO combined with FF (48.0 mg), PA (47.6 mg), and MAL (63.1 mg). Model compounds were dissolved in water (25 mL) and heated separately or as mixtures in sealed ampoules at 130 °C after adjusting the pH to 5.0 ± 0.1. At defined reaction times (0, 30, 60, and 120 min), samples were cooled down in a freezer to −20 °C to stop the reaction. Before further analysis, samples were centrifuged (10 min, 8175× *g*, 20 °C) and diluted, if necessary. Every sample was prepared in triplicate (all results are given as means ± standard de*via*tion).

### 2.3. Color Measurements 

The color of the aqueous extracts was characterized by L*a*b measurements as defined by *The International Commission on Illumination* (CIE) [40]. Visible spectra (350–800 nm) of the samples were recorded with a spectrophotometer (UV-1280, Shimadzu Deutschland GmbH, Duisburg, Germany; software: UV Probe Version 2.70). For illumination, a D65 lamp (Shimadzu) with an angle of 2° was used. The observer angle was set to 10°.

For characterization of the yellow to brown color typically formed in the Maillard reaction, additional measurements at 420 nm were conducted. Samples were diluted in water when the extinction exceeded 0.8.

All measurements were performed in a quartz cuvette against water.

### 2.4. HPLC-DAD Analysis of Furfural, Norfuraneol, Maltol, and Pyrrole-2-carbaldehyde

The conversion of FF, NF, MAL, and PA was quantified relative to the unheated samples (t = 0 min) by HPLC-DAD. Samples were diluted (1:20) and analyzed with the following setup: degasser, Shimadzu DGU-20As; pump, Shimadzu LC-20AD; autosampler, Shimadzu SIL-10AF; column oven, Shimadzu CTO-20A; column, Nucleosil^®^ 120-5 C18 (Macherey-Nagel GmbH & Co. KG, Düren, Germany); detector Shimadzu SPD-M20A; software, Shimadzu LabSolutions Version 5.90. The following settings were used: column temperature, 45 °C; flow rate, 1.0 mL/min; eluent A, 0.075% acetic acid in water (*v*/*v*); eluent B, methanol; eluent gradient, 0 min, 5% B; 10 min, 20% B; 15 min, 90% B; 20 min, 90% B; 21 min, 5% B; wavelength for quantitation, 285 nm.

### 2.5. HPLC-DAD Analysis of Methylglyoxal 

MGO was analyzed as its corresponding quinoxaline derivative and quantified relative to the unheated samples (t = 0 min) by HPLC-DAD [41]. For derivatization, aliquots of the model systems were incubated with *ortho*-phenylendiamine (OPD): 0.1 mL of OPD solution (50 mmol/L in water/methanol 1:1, *v*/*v*) was added to the sample (0.1 mL). Subsequently, samples were stored in darkness at room temperature for 24 h. After derivatization, samples were diluted (1:50) and analyzed by HPLC-DAD. The following setup was used: degasser, Shimadzu DGU-20As; pump, Shimadzu LC-20AD; autosampler, Shimadzu SIL-10AF; column oven, Shimadzu CTO-20A; column, Nucleosil^®^ 120-5 C18 (Macherey-Nagel GmbH & Co. KG, Düren, Germany); detector Shimadzu SPD-M20A; software, Shimadzu LabSolutions Version 5.90. The following settings were used: column temperature, 35 °C; flow rate, 0.5 mL/min; eluent A, 0.075% acetic acid in water (*v*/*v*); eluent B, methanol; eluent gradient, 0 min, 40% B; 10 min, 40% B; 15 min, 60% B; 25 min, 60% B; 26 min, 90% B; 34 min, 90% B; 35 min, 40% B; wavelength for quantitation, 318 nm.

### 2.6. Trolox Equivalent Antioxidant Capacity (TEAC) Assay of Aquoeus Solutions

An aqueous solution of ABTS (10 mmol/L) was incubated overnight at room temperature with an aqueous solution of potassium persulfate (3.5 mmol/L) for preparation of the radical cation solution. The working radical solution was prepared by diluting the radical cation solution 12:100 with phosphate buffer (5 mmol/L phosphate, pH 7.2–7.4) so that the absorbance of the blank at 734 nm was at 1.4 ± 0.02. Calibration was performed with six trolox standards (0.01, 0.02, 0.04, 0.06, 0.08, and 0.1 mmol/L; stock solution (10 mmol/L) was prepared with ethanol, standards were diluted in phosphate buffer). Then, 500 µL of the working solution and 500 µL of the samples (diluted in phosphate buffer) were mixed. Extinction at 734 nm was measured using a Biotek Uvikon XL (Agilent Technologies Inc., Santa Clara, CA, USA) after an incubation time of 120 min.

### 2.7. 2,2-Diphenyl-1-picrylhydrazyl Radical Assay of the Ethyl Acetate Extracts

The antioxidant activity of ethyl acetate extracts of the reaction mixtures was determined based on the capacity of the sample to reduce DPPH radicals. First, 200 µL of the aqueous reaction mixtures was extracted with 400 µL of ethyl acetate by shaking for 1 h. The two phases were separated and an aliquot of the ethyl acetate extract was diluted with methanol to be in the calibration range. DPPH was dissolved in methanol (1 mmol/L) and diluted in methanol until the absorbance of the blank (DPPH radical solution in methanol, 1:1 *v*/*v*) at 517 nm was at 1.4 ± 0.02. Calibration was performed with six trolox standards (0.01, 0.02, 0.04, 0.06, 0.08, and 0.1 mmol/L; diluted in methanol). Then, 500 µL of the DPPH radical solution and 500 µL of the diluted sample were mixed. After an incubation time of 120 min, extinction at 517 nm was measured in comparison to methanol using a Biotek Uvikon XL (Agilent Technologies Inc.). Every sample was prepared in triplicates (all results are given as means ± standard de*via*tion).

### 2.8. ESI(+)-Orbitrap Multiple-Stage High-Resolution Mass Spectroscopy

HRMS^n^ analyses were carried out as described before [41]: A Thermo Fisher Scientific Inc. LTQ Orbitrap XL™ instrument equipped with an Ion Max™ Source (Waltham, MA, USA) was used. Measurements were performed for each sample after heat treatment for 120 min and dilution (aqueous extracts diluted to 1 mg/mL in water; ethyl acetate extracts diluted to 0.5 mg/mL in ethyl acetate) in positive ion mode (ESI+) following direct infusion. Reserpine (0.05 mg/mL) was used for mass calibration. The normalized collision energy of collision induced dissociation varied from 5 to 50%. For the interpretation of the mass spectra, software Freestyle 1.6 was used (Thermo Fisher Scientific Inc.).

### 2.9. Statistical Analysis

All samples, aside from the qualitative SEC measurements, were prepared and analyzed in triplicates. Significant differences (*p* < 0.05) were identified by two-way analysis of variance (ANOVA) and Tukey’s test using GraphPad Prism 8.0.2 (San Diego, CA, USA).

## 3. Results and Discussion

### 3.1. Browning Potential and Color Formation

**Figure 2:**
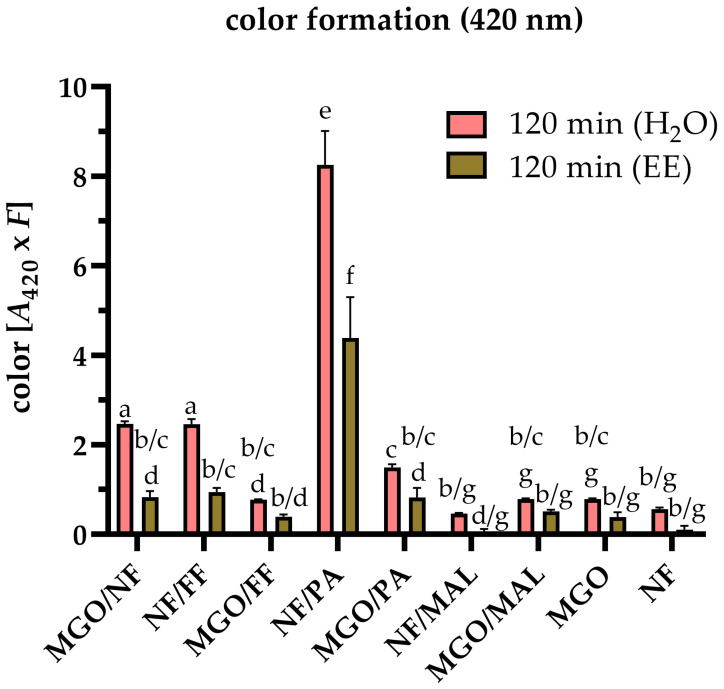
Browning intensity after thermal treatment of norfuraneol (NF) or methylglyoxal (MGO) individually as well as in combination with furfural (FF), pyrrole-2-carbaldehyde (PA), or maltol (MAL). Absorption at 420 nm of the aqueous reaction mixtures (red bars) and their ethyl acetate (EE) extracts (brown bars) after heating at 130 °C and pH 5 for 120 min. Statistical analyses were performed by two-way ANOVA and Tukey’s test (*p* < 0.05). Statistically equal values are designated by equal letters.

The analysis of color formation has been established as an useful method to characterize the reactivity of Maillard reaction systems [4,6,41,42,43,44]. For example, NF was found to form intensively colored oligomeric aldol reaction products, considered as melanoidin precursors (‘pre-melanoidins’), with different carbohydrate-derived carbonyl compounds such as glycolaldehyde [6], glyoxal [6], MGO [41], and FF [7], as well as nitrogen-containing intermediates, such as PA [7]. NF was also reported to form a reactive triketone following oxidation [43]. This triketone contributes to the formation of heterogenous, colored oligomers in multiple ways: It could be directly involved in the oligomerization by aldol reactions, but it can also contribute indirectly to the color formation after its cleavage, yielding reactive short-chain carbonyls that partake in subsequent reactions [6]. However, less is known about the reactivity of MGO with electrophilic carbonyl compounds and the role of the antioxidant reductone ether MAL in non-enzymatic browning reactions. To gain a deeper understanding of the reactivity of different groups of Maillard reaction intermediates, the color formation after incubating binary reaction mixtures of NF or MGO with FF, MAL, and PA as well as of the individual reactants was analyzed by Vis spectroscopy after heat treatment at 130 °C and pH 5 for 120 min. Additionally, the amount of apolar colorants was assessed by color measurements after extracting the reaction mixtures with ethyl acetate. Figure 2 shows the absorbance at 420 nm of the aqueous reaction mixtures (red bars) after 120 min of heating and their ethyl acetate extracts (brown bars). The ratio of ethyl acetate to water of 2:1 was considered by multiplying the absorbance of the ethyl acetate extracts by 2. The absorbance of the colorless starting solutions (Figure S1A; t = 0 min) as well as of individually treated FF, MAL, and PA (Figure S1B, t = 120 min) were negligible and are shown in the Supplementary Material.

Heat treatment at 130 °C and pH 5 for 120 min resulted in elevated browning for all binary aqueous model systems as well as for individually treated NF and MGO. However, only the color formation of MGO/NF, NF/FF, and NF/PA could be interpreted as synergistic, because color measurements of these binary systems revealed significantly higher absorbances compared to the sums obtained after incubation of the individual reactants by 83%, 272%, and 1355%, respectively. The browning intensity of NF/PA was around four times higher compared to NF/FF as well as MGO/NF. The increased reactivity of PA compared to FF could be explained by the lower electronegativity of nitrogen compared to oxygen resulting in an increased electron density of the corresponding aromatic carbon atoms. This results in a higher reactivity of PA as a nucleophile [45], for example, with the carbonyl function of NF, its cleavage products [6], and α,β-unsaturated aldol condensation products by Michael addition [7], overall leading to a faster formation of colored reactions products. Further, the additional reactive center of PA is hypothesized to enable the formation of larger chromophores by crosslinking reactions.

Similar results were found for MGO, as the browning of MGO/PA tended to be higher compared to MGO/FF and MGO. However, among the MGO reaction systems, MGO/NF exhibited the most intense browning following thermal treatment. Based on the higher nucleophilicity of NF compared to MGO, it is assumed that MGO primarily reacts as an electrophile in this reaction. MGO/FF, MGO/MAL, and NF/MAL exhibited significant browning, but it was comparable to the individual browning reaction of MGO. As the browning intensity after treatment of MGO was slightly higher compared to NF, it was assumed that the thermally induced cleavage of NF into short chain carbonyls, including MGO [3,6], precedes the observed color formation, which delays the formation of colored oligomers in comparison to MGO. 

Generally, the colorants soluble in ethyl acetate accounted for around half of the total color values measured in aqueous reaction mixtures. Only the ethyl acetate extracts of NF/MAL and NF were colorless. This seems to be plausible, because the aqueous conditions favor the formation of polar aldol addition products [6]. The formation of apolar products could be attributed to aldol condensation reactions which were already reported for NF/PA [7], NF/FF [7], and MGO/NF [41], but not for MGO/FF, MGO/PA, and individually treated MGO. The antioxidant reductone ether MAL was the only compound that was not associated with elevated browning intensities, neither individually nor in combination with MGO or NF, regardless of the solvent used.

**Figure 3:**
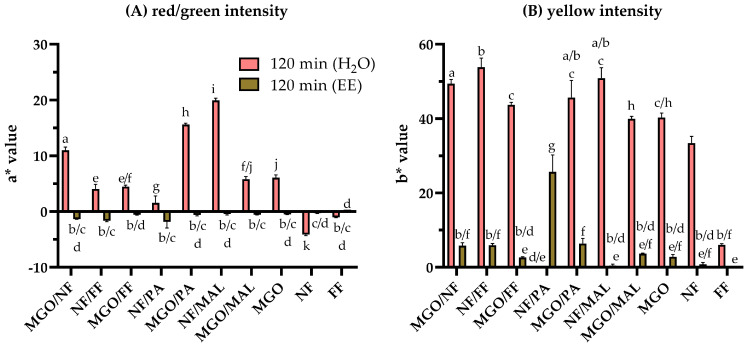
Color intensity of the reaction mixtures resulting from thermal treatment of norfuraneol (NF) or methylglyoxal (MGO) in combination with furfural (FF), pyrrole-2-carbaldehyde (PA), or maltol (MAL) at 130 °C and pH 5 for 120 min. (**A**) Red/green intensity (a* value) and (**B**) yellow/blue intensity (b* value) of the aqueous reaction mixtures (red bars) and their ethyl acetate extracts (olive brown bars) for the MGO model systems and the NF model systems. Statistical analyses were performed by two-way ANOVA and Tukey’s test (*p* < 0.05). Statistically equal values are designated by equal letters.

To complement the characterization of the colorants formed in the model systems after heat treatment, the color values a* (red/green intensity) and b* (yellow/blue intensity) were analyzed and are shown in Figure 3A and Figure 3B, respectively. The lightness (L*) of the samples (aqueous solutions, Figure S2A; ethyl acetate extracts, Figure S2B), the a* (Figure S3A) and b* values (Figure S3B) of the initial solutions (t = 0 min) as well as those of PA and MAL after 120 min of thermal treatment (a* value, Figure S3C; b* value, Figure S3D) are shown in the Supplementary Material.

Initial lightness of all reaction mixtures (Figure S2A, Supplementary Material) was around 100% and heat treatment resulted in an inverse correlation between lightness and browning intensity at 420 nm of the aqueous reaction mixtures. 

Heat treatment for 120 min induced significant changes to the color intensities. The initial a* value of NF and FF was highly reduced compared to the starting solutions. Except for NF and FF, the aqueous solutions exhibited an increased red intensity with a* values in a range between 2 and 20 (red bars, Figure 3A). a* was highest for NF/MAL (19.9), MGO/PA (15.6), and MGO/NF (11.0), intermediate for MGO (6.1), MGO/MAL (5.8), MGO/FF (4.5), and NF/FF (4.0), and lowest for NF/PA (1.6). Even though the browning intensity of NF did not change after addition of MAL, combined treatment of NF/MAL resulted in a significantly higher a* value compared to the individual reactants, indicating that the presence of MAL impacted the pathways of the browning reaction and shifted the product spectrum to red chromophores. Because of the high color intensity (low L* values) of the aqueous NF/PA reaction mixture, it was not possible to qualify the color quality after 120 min resulting in low a* and b* values.

Extraction of the aqueous mixtures in ethyl acetate (Figure 3A, brown bars) resulted in negligible values for a* indicating that the colorants exhibiting a red or green color were not soluble in ethyl acetate. The red color of MAL and PA was negligible after thermal treatment, independently of the solvent (Figure S3C, Supplementary Material).

Analyses of the yellow intensity (Figure 3B) revealed that predominantly yellow colorants were soluble in ethyl acetate. All the investigated binary aqueous reaction mixtures (red bars) and individually treated MGO were characterized by b* values between 40 and 50. The yellow intensity of individually treated NF (33) and FF (6) were significantly lower. The yellow color of MAL and PA was negligible after thermal treatment, independently of the solvent (Figure S3D, Supplementary Material).

The b* values of the ethyl acetate extracts (brown bars, Figure 3B) largely resemble the browning intensity determined at 420 nm (brown bars, Figure 2). As the red intensity was negligible, it could be assumed that the browning originated from predominantly apolar, yellow colorants, presumably aldol condensation products.

### 3.2. Heat-Induced Conversion of the Maillard Reaction Intermediates

To evaluate the participation of the different Maillard reaction intermediates in the formation of the colorants characterized before, the conversion of the reactants was analyzed by HPLC.

**Figure 4:**
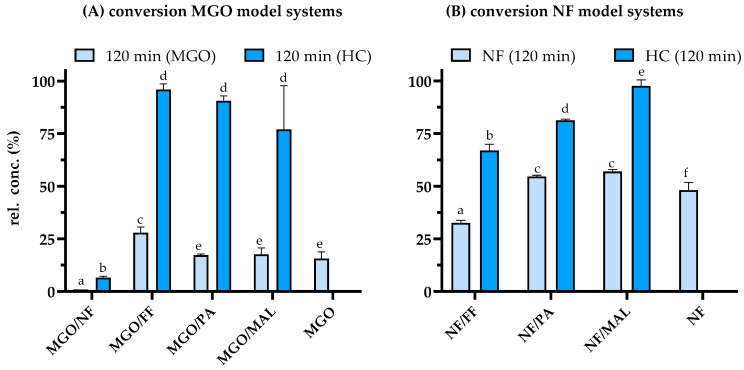
Reactivity of the binary mixtures composed of methylglyoxal (MGO) or norfuraneol (NF) with the heterocyclic compounds furfural (FF), pyrrole-2-carbaldehyde (PA), and maltol (MAL). Conversion of (**A**) the MGO reaction systems (light blue bars; heterocyclic (HC) reactants, blue bars) and (**B**) the NF reaction systems (light blue bars; heterocyclic reactants, blue bars) after heat treatment at 130 °C and pH 5 for 120 min. Statistical analyses were performed by two-way ANOVA and Tukey’s test (*p* < 0.05). Statistically equal values are designated by equal letters.

Generally, heat treatment of MGO (Figure 4A) led to a high conversion of the 1,2-dicarbonyl compound (light blue bars). Individual incubation of MGO resulted in a conversion of 84% and comparable values were obtained for MGO/PA and MGO/MAL. The highest and lowest relative concentration of 28% and 1% were determined after the heat treatment of MGO/FF and MGO/NF, respectively. MGO/NF were also reported previously as a highly reactive mixture, inducing an almost complete conversion of MGO after 60 min of treatment under the same conditions [41]. In contrast to the high turnover of MGO, the relative concentration of FF, PA, and MAL in the binary model systems did not significantly decrease (blue bars, Figure 4A), but the mean values showed the tendency of an increasing conversion of 4%, 9%, and 23%, respectively. Thus, the conversion of the binary model systems differed from those obtained after incubation of the individual heterocyclic compounds (Figure S4, Supplementary Material). NF was characterized by the highest conversion after incubation with MGO as only 7% of the initial amount used could be quantified after heat treatment of MGO/NF (Figure 4A). In contrast, around 48% of NF was left after its individual treatment which demonstrates its increased reactivity when incubated with MGO (light blue bars, Figure 4B). 

Considering the conversion of the reactants in the MGO model systems, NF was the only heterocyclic compound (HC) to significantly increase the non-enzymatic browning of MGO resulting in a synergistic color formation. The higher MGO content at the end of the thermal treatment in the MGO/FF system indicates that FF inhibited the conversion of MGO, without impacting the browning intensity of MGO/FF compared to MGO. Consequently, the yield of colored products relative to the MGO turnover was significantly higher for MGO/FF.

The observed conversion rates of the reactants in all binary systems showed that the underlying reactions are not simple stochiometric reactions of both reactants. In all cases, the nucleophilic compounds in form of MGO or NF exhibited a faster conversion than the heterocyclic aldehydes indicating that MGO and NF undergo subsequent reactions with themselves or their degradation products. Both compounds offered more attractive electrophilic centers than the heterocycles FF, MAL, or PA. Reactions based on electrophilic aromatic substitution reactions seem to be of minor importance under the given reaction conditions. The incubation of MGO/NF was characterized by the highest conversion of both reactants resulting in synergistic color formation. Due to the conjugated system of NF, the resulting chromophore systems of these heterogenous, potentially aromatic, reaction products were larger compared to potential MGO oligomers, being reflected in the increased browning intensity of MGO/NF compared to individually treated MGO (Figure 2).

Compared to the MGO systems, the NF/PA and NF/MAL systems exhibited a lower conversion rate of NF yielding a conversion rate of approximately 45%. The relative end concentration of 55% was significantly higher compared to the 48% NF that remained after its individual treatment. A higher conversion rate of NF was determined for NF/FF to about 33% of the initially used amount. Except for MAL, the combined incubation of the NF model systems resulted in a significant conversion of its heterocyclic reaction partners, as well. Thus, around 81% and 67% of the initially used amount of PA and FF were quantified after incubation of NF/PA and NF/FF, respectively. An even higher conversion of both HCs, namely NF and FF, occurred after their incubation under dry conditions at 100 °C. After 60 min, NF was fully converted and only 40% of the initially used amount of FF was quantified [7]. This comparison indicates that aldol condensation reactions, which are favored under dry conditions, are preferred for NF with conjugated carbonyl compounds as they result in the formation of even larger conjugated reaction products.

Even though NF/PA showed a lower conversion rate than NF/FF, the color formation was considerably higher (Figure 2). Overall, this system exhibited the strongest color formation relative to the consumed reactants, even in comparison to MGO/NF. These findings demonstrate that there is no strict correlation between the conversion of the reactants and the browning intensity. Even when a lower conversion of the reactants correlates with a lower yield of colored products taking place, the color intensity of the resulting Maillard reaction systems might depend on the extinction coefficient of individual products rather than their quantity.

### 3.3. Molecular Weight Distribution of Colored Reaction Products

**Figure 5:**
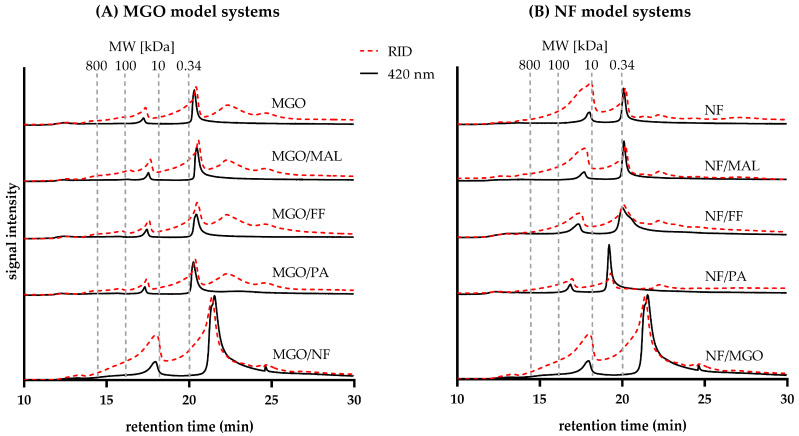
Molecular weight distribution of the binary aqueous reaction mixtures composed of methylglyoxal (MGO) or norfuraneol (NF) with the heterocyclic compounds furfural (FF), pyrrole-2-carbaldehyde (PA), and maltol (MAL). SEC analysis of the different reaction systems obtained from (**A**) MGO and (**B**) NF after heating at 130 °C for 120 min (pH 5). Detection was performed at 420 nm (black line) and *via* a refractive index detector (red-dashed line).

SEC was used to characterize the molecular weight distribution of the reaction products formed after heat treatment of the selected Maillard intermediates for 120 min (Figure 5). Colored fractions were identified based on their absorbance at 420 nm (black line). Refractive index detection (RID, red-dashed line) was used to estimate the amount of all substances present in the analyzed reaction mixtures.

Analysis of MGO samples (Figure 5A) revealed the formation of two specific molecular weight domains of colored products: one low-molecular-weight fraction co-eluting with the sucrose standard (0.34 kDa) at 21.0 min and a second fraction at 17.5 min with a higher molecular weight, eluting between the 10 kDa and 100 kDa standards. The intensity of the RID signal and the absorbance at 420 nm were almost equal for the fraction eluting at 21.0 min, whereas the absorbance at 420 nm was lower relative to the RID signal for the fraction exhibiting the higher molecular weight. Overall, the low-molecular-weight fraction accounted for the larger proportion of compounds in the reaction mixture. Additionally, the contribution of the low-molecular-weight fraction to the browning intensity of the reaction mixture was significantly higher. Apart from these colored domains, there were two colorless fractions, only visible by RID, eluting after the sucrose standard, which might be attributed to MGO at 25 min and potentially small oligomers of MGO (e.g., its dimer or trimer) eluting at 22.5 min.

The size-exclusion chromatograms of the mixtures MGO/MAL, MGO/FF, and MGO/PA did not show any considerable differences compared to MGO, which is a further indication that MAL, FF, and PA did not relevantly partake in the browning reaction of MGO. Further, the individually treated HCs were not found to yield any colored products, as well (Figure S5, Supplementary Material).

SEC analysis of MGO/NF revealed the formation of three molecular domains of colored reaction products that differed from those of MGO: a high-molecular-weight fraction was eluting at around 18.0 min shortly after the 10 kDa standard. The signal intensity of its RID signal was around twice as high as the absorbance at 420 nm. The second fraction accounted for the highest amount of compounds in the mixture eluting at 22.0 min with comparable intensities of the RID signal and absorbance at 420 nm. This molecular domain could be composed of small aldol reaction products, formed from both reactants. The third fraction was a small and sharp peak around 24.5 min, exhibiting the lowest RID intensity and absorbance at 420 nm which might be a precursor of the fraction eluting at 21.0 min. Previously, it was shown that longer heating times of MGO/NF (300 min) resulted in the formation of colorants with comparably higher molecular weights and two distinct domains around 100 kDa and between 0.34 kDa and 10 kDa [6]. This shows that colorants formed after 120 min of heat treatment can undergo subsequent reactions leading to the formation of even larger colorants.

The molecular domains detected by SEC analysis of NF and NF/MAL (Figure 5B) were almost identical: one high-molecular-weight fraction was eluting around 18.0 min, coeluting with the 10 kDa standard, and exhibited a significantly higher RID signal intensity compared to its low absorbance at 420 nm. The fraction eluting at 20 min (around 0.34 kDa) accounted for the largest quantity in the reaction mixture. Its RID signal and the absorbance at 420 nm exhibited almost equal intensities. Another signal, only visible by RID, eluted at 22.5 min and could originate from the reactants. Comparable findings resulted for NF/FF with some differences: both colored fractions eluted later, suggesting a higher molecular weight. The absorbance at 420 nm for the fraction eluting at approximately 17.8 min (10 kDa) was notably higher, with only a marginal decrease in comparison to its RID signal intensity. In addition, the peak of the low-molecular-weight fraction at approximately 20 min (around 0.34 kDa) was comparatively broad, implying the formation of a higher amount and variety of colored reaction products. The slightly increased molecular weight and the elevated abundance of colored products could explain the intensified browning observed for NF/FF compared to NF and NF/MAL (Figure 2). In earlier investigations using longer incubation times (300 min), heat treatment of NF/FF resulted in the formation of two distinct domains, as well. In contrast to the present findings, those two colored fractions exhibited a higher molecular weight of 100 kDa and 10 kDa [6]. This is another indication that in the course of heat treatment, low-molecular weight colorants can undergo subsequent reactions yielding high-molecular weight and colored reaction products.

The SEC analysis of NF/PA differed significantly from the other mixtures and two colored molecular domains were detected: one fraction with a comparatively low signal intensity at 16.0 min, eluting between the 10 and 100 kDa standard, as well as a dominant fraction eluting between the 0.34 kDa and 10 kDa standards at 19.0 min. The latter exhibited a high browning intensity and the absorbance at 420 nm exceeded the signal intensity of the RID by a factor of two. One low-molecular-weight fraction eluted at 22.5 min, only visible by RID, which could be attributed to the reactants.

SEC showed that predominantly small molecules eluting between the 0.34 kDa and 10 kDa standards were found to significantly contribute to the browning intensity of all the investigated reaction mixtures, whereas fractions with a higher molecular weight between 10 kDa and 100 kDa exhibited a comparatively low absorbance at 420 nm. In contrast to earlier investigations of melanoidins formed by thermal treatment of 3-deoxyglucosone with γ-aminobutyric acid [5] and MGO with l-alanine [4], whose intense browning was attributed to colorants with a molecular weight significantly above 100 kDa, the model experiments investigated in the present study primarily yielded smaller colorants, supposedly melanoidin precursors or pre-melanoidins. The system NF/PA demonstrates the browning potential of nitrogen-containing Maillard intermediates leading to the formation of high-molecular-weight melanoidins.

### 3.4. Antioxidant Properties of the Colored Reactions Products

Melanoidins are discussed frequently in terms of their antioxidant activity, with different findings on the relationship between the molecular size, the color intensity, and the antioxidant activity, depending on reactants involved in the melanoidin formation. For instance, the antioxidant activity of heated honey [46] was mainly attributed to water-soluble high-molecular-weight melanoidins. The authors reported that the color of the melanoidin fractions positively correlated with their molecular weight (measured by SEC), as well. In detail, the antioxidant activity and the color of a fractions assigned to a molecular weight of 180–232 kDa and 140–180 kDa was higher compared to lower-molecular-weight fractions with 109–140 kDa and 85–109 kDa. On the other hand, Del Castillo et al. [28] reported that the high antioxidant activity of roasted coffee extracts was predominantly caused by low-molecular-weight components (3.5–14.7 kDa), which exhibited a significantly higher antioxidant activity but less intense color compared to melanoidins from dark roasted coffee extracts (>14.7 kDa). 

**Figure 6:**
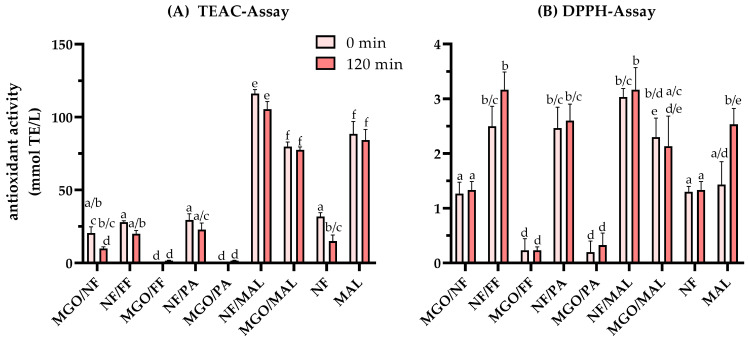
Antioxidant properties of the reaction mixtures after thermal treatment of norfuraneol (NF) or methylglyoxal (MGO) in combination with furfural (FF), pyrrole-2-carbaldehyde (PA), or maltol (MAL). (**A**) Antioxidant activity of the aqueous reactions mixtures obtained by TEAC and (**B**) their ethyl acetate extracts obtained by DPPH assay in comparison to trolox before thermal treatment (light red bars) and after heat treatment at 130 °C and pH 5 for 120 min (red bars). Statistical analyses were performed by two-way ANOVA and Tukey’s test (*p* < 0.05). Statistically equal values are designated by equal letters.

To characterize the influence of different Maillard reaction intermediates on the antioxidant activity of the colorants formed after heat treatment, TEAC (Figure 6A) and DPPH (Figure 6B) assay were performed for the aqueous model systems and their ethyl acetate extracts, respectively.

Overall, the antioxidant activity determined by the TEAC assay of the aqueous solutions were higher compared to ethyl acetate extracts obtained by the DPPH assay, despite the different volume of the extraction agent considered. However, the relative trend between the investigated model systems was different.

The highest antioxidant activity of 116 mmol TE/L was determined for the aqueous starting solutions (light red bars, Figure 6A) of NF/MAL (20 mmol/L, each), which did not decline significantly (*p* < 0.05) to 106 mmol TE/L after thermal treatment for 120 min (red bars). The comparatively high antioxidant activity of this binary mixture could be attributed to the individual components MAL and NF with antioxidant activities of 88 mmol TE/L and 31 mmol TE/L, respectively. Comparable to the non-significant conversion (Figure 4), thermal treatment of MAL did not result in a significant change of its antioxidant activity, whereas heat-induced reactions of NF resulted in a significant decline of NF’s antioxidant activity by approximately 50%. As the antioxidant activity of MAL was almost threefold compared to NF, only a non-significant (*p* < 0.05) reduction of the antioxidant activity could be observed in the mixture of NF/MAL. The antioxidant activity of the NF/PA, NF/FF, and MGO/NF starting solutions were comparable to those of NF. Heat treatment did not result in significant changes of the antioxidant activity of NF/PA and NF/FF. This demonstrates that the formation of at least partially conjugated products with preserved NF units [7] results in colored reaction products with antioxidant properties comparable to those of native NF. Heat treatment induced a significant decline of the antioxidant activity of MGO/NF to around 50% of the starting solutions. Considering the heat-induced cleavage reactions of NF to short chain carbonyls [3,6], the loss of the antioxidant activity of NF and MGO/NF implies that the polar colorants formed by NF with short chain carbonyls exhibit significantly lower antioxidant activity compared to native NF. This could be explained by the predominant formation of aldol addition products under aqueous conditions with isolated π-electron systems between the NF monomers [6]. A higher antioxidant activity would be expected from condensation products, which should be able to stabilize radicals in conjugated π-electron systems.

Apart from MGO/NF and MGO/MAL, whose elevated antioxidant activities can be attributed to NF and MAL, respectively, the aqueous model mixtures MGO/FF and MGO/PA, as well as the mixtures obtained after individual treatment of FF, PA, and MGO (Figure S6, Supplementary Material) did not show a considerable antioxidant activity. 

The DPPH assay of the ethyl acetate extracts (Figure 6B) yielded generally significantly lower antioxidant activities compared to the corresponding aqueous solutions. However, the change of the antioxidant activity induced by non-enzymatic browning reactions gives insight into the contribution of less polar reaction products to the antioxidant activity of the respective model systems. 

The highest antioxidant activity was observed for the combinations of NF/FF, NF/PA, and NF/MAL. However, there was no significant difference between the antioxidant activity of the ethyl acetate extracts of the starting solution and those of the heated mixtures. The initial antioxidant activities obtained for NF/FF, NF/PA, and NF/MAL could be attributed to the sum of the individual compounds. Only the thermal treatment of NF/FF showed the tendency to result in the formation of colored products with an increased antioxidant activity, but this change was not significant compared to the starting solutions. It is of note that, even though NF was converted to a higher extent, the antioxidant activity of NF/FF did not significantly change throughout the thermal treatment. This indicates that the colored condensation products were composed of preserved NF units, as well.

The antioxidant activity of MGO/NF was comparable to NF. In contrast to the decline of the antioxidant activity of the aqueous solutions determined by TEAC, the heat treatment did not result in significant changes of the antioxidant activity of the ethyl acetate extracts. This implies that the formation of apolar condensation products soluble in ethyl acetate indeed results in preserving the antioxidant properties of NF. The DPPH assay of MGO/FF (0.23 mmol TE/L) and MGO/PA (0.2 mmol TE/L) revealed a comparatively low antioxidant activity, regardless of a thermal treatment.

Among the individual reactants, the highest antioxidant activity was assigned to NF and MAL. Initially, both were around 1.3 mmol TE/L. In contrast to the aqueous reaction mixture of MAL, DPPH assay revealed an increase of the antioxidant activity of around 1.1 mmol TE/L for the heated reaction mixture. As there were no significant heat-induced changes in the concentration of MAL, this increase could have resulted from some minor reactions of MAL, leading to more intense antioxidants soluble in ethyl acetate. In comparison, the antioxidant activity of the ethyl acetate extracts obtained after extraction of the heated NF reaction mixture did not change.

### 3.5. Structural Composition of the Antioxidant Pre-Melanoidins

To understand the antioxidant properties of the pre-melanoidins formed in the different reaction mixtures, HRMS after direct injection were applied for structural characterization. HRMS spectra of the aqueous reaction mixtures obtained after 120 min of heat treatment and the corresponding ethyl acetate extracts were recorded *via* direct infusion, both in positive and negative ion mode using electron spray ionization (ESI). ESI+ of the ethyl acetate extract provided the best ion intensities with spectra, allowing to understand antioxidant properties of selected reaction products based on their proposed structures. The lower ion yield of the aqueous reaction solutions might be due to the comparatively high concentration of Na^+^ and Cl^-^ resulting from the initial adjustment of the pH value. 

**Figure 7:**
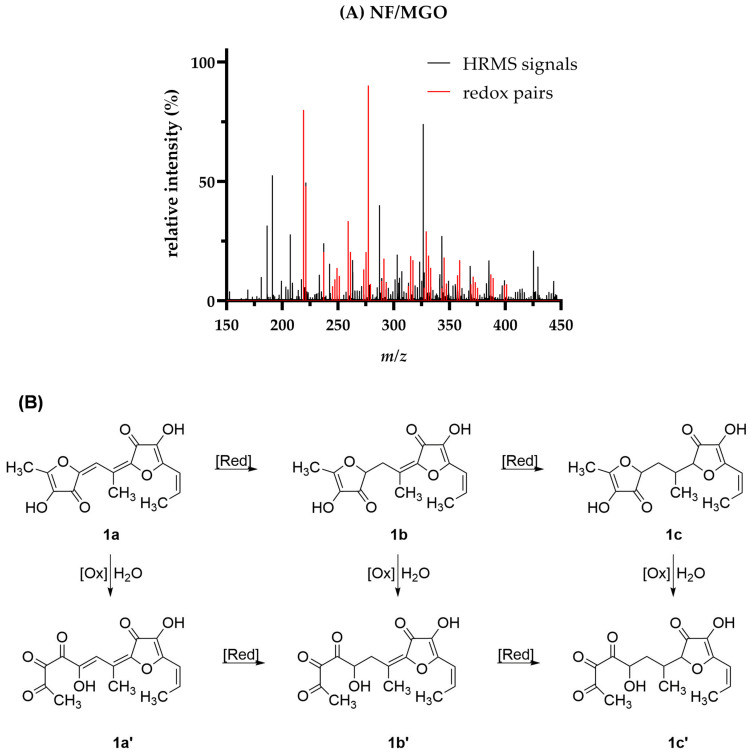
HRMS analysis and structural proposal for the antioxidant reaction products formed after treatment of methylglyoxal/norfuraneol (MGO/NF). (**A**) HR-ESI(+)-MS spectrum of the ethyl acetate extracts and (**B**) proposed redox mechanism for a reaction product 1 and its oxidized derivatives 1’ assigned to a condensation product of two units NF, MGO, and acetaldehyde (AA).

The HRMS scan spectrum of MGO/NF is shown in Figure 7A (black lines). The assignment of the molecular formulae was based on the exact masses of the detected ions. *Via* the sum formula, it was possible to assign a tentative composition based on the used reactants and their relevant cleavage products. More precisely, acetaldehyde (AA) and formaldehyde were identified as relevant degradation products of NF [6] and MGO [47]. Apart from the reactants and their degradation products, condensation (–H_2_O), redox reactions (±H_2_), and the hydrolytic, oxidative cleavage of NF (+O) were considered, as well. A detailed discussion about the formation mechanism and the structural composition of these aldol and Michael products has been published recently [4,6,7,41]. 

Two NF molecules can be linked *via* a condensed bridge after condensation with MGO. The C-C-double bonds of the former MGO unit might undergo two separate reduction steps to compounds **1b** (*m*/*z* 315) and **1c** (*m*/*z* 317), both detected by HRMS. Additionally, the NF unit may also perform an oxidation after its hydrolytic cleavage [3], which is indicated by the formal addition of one oxygen atom to the sum formula. This mechanism is proposed for the redox pairs **1b**/**1b’** (*m*/*z* 315 and *m*/*z* 331) and **1c**/**1c’** (*m*/*z* 317 and *m*/*z* 333). As the discussed redox pairs of MGO/NF were predominantly detected as formally ‘redox native’ (number of oxidation reactions equal reduction reactions) and reduced (number of formal reduction reactions is higher compared to oxidation reactions) species, it was assumed that these reaction products do not exhibit a high reducing power and therefore did not contribute to the antioxidant activity to a comparable extent to NF. Alternatively, **1b’** and **1c’** could be also the hydrated species of **1a** and **1b**, respectively, as HRMS analyses do not allow to differentiate these isomers. The prevalence of oxidized NF (+ O) subunits is more explicit for the assignment of species in the colored mixtures of NF/PA, NF/FF, and NF.

The decreased reducing power of the reaction products formed by incubation of MGO/NF is also reflected by the TEAC results, as the antioxidant activity of MGO/NF significantly declined after thermal treatment. Analogue findings were made after HRMS analyses of MGO, MGO/MAL, and MGO/FF, in which no oxidation of the native compounds was detected, but only various degrees of reduction (Table 1 and Figure S7, Supplementary Material). 

Nevertheless, under harsher conditions, for example food roasting or in food matrices containing strong oxidizing agents, these compounds might still react as reducing agents, resulting in their oxidation. The oxidation of the discussed heterogenous compounds was proposed by Kanzler and Haase [7] to result in an enlargement of the conjugated π-electron system and finally, the intensification of the color. It was hypothesized that such reactions underlie the increased antioxidant activity and browning intensity of NF/FF, NF/PA, and NF, which in parallel showed a high prevalence of heterogenous, oxidized condensation products of the respective reactants within the redox clusters (Table 1 and Figure S7). These findings indicate that the colored pre-melanoidins formed by condensation reactions of heterocyclic Maillard intermediates exhibit antioxidant properties, which might derive from the resonance stabilization throughout an enlarged conjugated system. In contrast, pre-melanoidins composed of short chain aliphatic carbonyls do not significantly contribute to the antioxidant activity. 

HRMS analyses of MGO/PA and NF/MAL did not result in the detection of signals that could be reasonably assigned to analogue redox pairs.

## 4. Conclusions

In the present study, the heat-induced reactivity of the known color precursors NF and MGO with other prominent Maillard reaction intermediates was investigated to characterize the antioxidant activity, the molecular weight distribution, and the browning intensity of potential melanoidin precursors depending on the reactants involved. SEC analysis demonstrated that primarily small reaction products with a molecular weight between 0.34 kDa and 10 kDa were responsible for the brown color of the different reaction mixtures. This allowed an in-depth characterization of pre-melanoidins, the early-stage colored precursors of the heterogenous high-molecular-weight end-products of the Maillard reaction.

The model experiments revealed that only the reaction of NF with electrophilic carbonyls, especially PA, resulted in synergistic color formation. Based on the conversion of the reactants, it was demonstrated that the color yield per mol of the reactants was multiples higher when NF was incubated with heterocyclic aldehydes instead of the short-chained α-dicarbonyl MGO.

Both the concentration of NF and the antioxidant activity of the reaction mixture obtained after its individual heat treatment were reduced by 50% in comparison to the starting solution. Although NF was converted to a comparable extent after incubation with FF and PA, the antioxidant activity of native NF was almost fully preserved in the colored reaction mixtures, implying that it retained its ability to react as an antioxidant when incorporated into conjugated chromophore structures. The antioxidant activity of the corresponding reaction products of NF was verified by the detection of different redox clusters by means of HRMS.

Opposing findings for the reaction mixtures of MGO were obtained. Independent of the reaction partner, MGO exhibited the highest reactivity based on its conversion, without resulting in the highest browning intensity. Additionally, the colored reaction mixtures were not found to exhibit an increased antioxidant activity compared to the reactants. Even in combination with NF, the antioxidant activity declined by around 50%. 

In conclusion, this study gives fundamental insights into the early stage of color-forming reactions occurring during the Maillard reaction. It is the first to show that the antioxidant properties of melanoidins could result from the incorporation of conjugated, antioxidant intermediates whose properties can be preserved in heterogenous colored products, whereas the formation of new antioxidant structures could not be determined in this study. This knowledge is highly relevant to understand the contribution of individual Maillard reaction on the properties of melanoidins formed in thermally treated food and help to proceed in the process of unraveling the structure of these heterogenous, complex high-molecular-weight compounds. Further, this study shows the origin of antioxidant compounds formed during food processing that significantly contribute to the conservation and the shelf-life of food. These processing-borne and heat-resistant antioxidants complement natural food antioxidants, for example phenolic compounds, whose antioxidant properties are sometimes lost due to thermally induced conversion. For product development and optimization, antioxidant Maillard intermediates can be used to synthesize comparatively stable antioxidant colorants that fulfil the requirements of two different groups of food additives—colorants and antioxidants. Finally, the antioxidant pre-melanoidins and melanoidins characterized in the present study are key components in human nutrition and food processing that significantly improve food quality and the compatibility of food.

## Figures and Tables

**Table 1:** Selection of signals assigned to redox active products detected in the ethyl acetate extracts of MGO/NF (Figure 7), MGO/FF, NF/FF, MGO/MAL, NF/PA, MGO, and NF (Figure S6). Oxidation was reflected by a loss of hydrogen (–H_2_) and the addition of oxygen (+O). Reduction was implied by the addition of hydrogen (+H_2_).

	Structure Assignment								
Compounds			H_2_O	H_2_	O	Composition	Exp.	Theo.	Rel. Error (ppm)
MGO/NF									
2 × NF	1 × MGO	1 × AA	–3	0	0	C_15_H_14_O_6_Na^+^	313.0687	313.0683	1.44
2 × NF	1 × MGO	1 × AA	–3	1	0	C_15_H_16_O_6_Na^+^	315.0844	315.0839	1.41
2 × NF	1 × MGO	1 × AA	–3	2	0	C_15_H_18_O_6_Na^+^	317.1000	317.0996	1.42
2 × NF	1 × MGO	1 × AA	–3	1	1	C_15_H_16_O_7_Na^+^	331.0793	331.0788	1.50
2 × NF	1 × MGO	1 × AA	–3	2	1	C_15_H_18_O_7_Na^+^	333.0950	333.0945	1.53
MGO/FF									
2 × FF	2 × MGO	1 × AA	–2	0	0	C_18_H_16_O_7_Na^+^	367.0793	367.0788	1.24
2 × FF	2 × MGO	1 × AA	–2	1	0	C_18_H_18_O_7_Na^+^	369.0949	369.0945	1.19
2 × FF	2 × MGO	1 × AA	–2	2	0	C_18_H_20_O_7_Na^+^	371.1106	371.1101	1.25
NF/FF									
2 × NF	1 × FF	-	–1	– 1	0	C_15_H_12_O_7_Na^+^	327.0479	327.0475	1.17
2 × NF	1 × FF	-	–1	0	0	C_15_H_14_O_7_Na^+^	329.0634	329.0632	0.76
2 × NF	1 × FF	-	–1	1	0	C_15_H_16_O_7_Na^+^	331.0792	331.0788	1.27
2 × NF	1 × FF	-	–1	2	0	C_15_H_18_O_7_Na^+^	333.0952	333.0945	2.32
2 × NF	1 × FF	-	–1	–1	1	C_15_H_12_O_8_Na^+^	343.0428	343.0424	1.08
2 × NF	1 × FF	-	–1	0	1	C_15_H_14_O_8_Na^+^	345.0586	345.0581	1.34
2 × NF	1 × FF	-	–1	1	1	C_15_H_16_O_8_Na^+^	347.0742	347.0737	1.25
2 × NF	1 × FF	-	–1	2	1	C_15_H_18_O_8_Na^+^	349.0897	349.0894	0.82
MGO/MAL									
1 × MAL	1 × MGO	1 × AA	–2	1	0	C_14_H_16_O_6_Na^+^	303.0845	303.0839	1.95
1 × MAL	1 × MGO	1 × AA	–2	2	0	C_14_H_18_O_6_Na^+^	305.1002	305.0996	2.01
NF/PA									
2 × NF	1 × PA	-	–1	– 1	0	C_15_H_13_O_6_NNa^+^	326.0636	326.0635	0.28
2 × NF	1 × PA	-	–1	0	0	C_15_H_15_O_6_NNa^+^	328.0794	328.0792	0.85
2 × NF	1 × PA	-	–1	–1	1	C_15_H_13_O_7_NNa^+^	342.0581	342.0584	– 0.93
2 × NF	1 × PA	-	–1	0	1	C_15_H_15_O_7_NNa^+^	344.0741	344.0741	0.07
MGO									
5 × MGO		-	–4	1	0	C_15_H_14_O_6_Na^+^	313.0688	313.0683	1.6
5 × MGO		-	–4	2	0	C_15_H_16_O_6_Na^+^	315.0844	315.0839	1.55
5 × MGO		-	–4	3	0	C_15_H_18_O_6_Na^+^	317.1001	317.0996	1.56
NF									
3 × NF		-	–1	–1	0	C_15_H_14_O_8_Na^+^	345.0585	345.0581	1.25
3 × NF		-	–1	0	0	C_15_H_16_O_8_Na^+^	347.0742	347.0737	1.21
3 × NF		-	–1	0	1	C_15_H_16_O_9_Na^+^	363.0690	363.0687	0.94
3 × NF		-	–1	0	1	C_15_H_16_O_10_Na^+^	379.0639	379.0636	0.78

## Data Availability

The data presented in this study are available on request from the corresponding author.

## Supplementary Materials

The following supporting information can be downloaded at: https://www.mdpi.com/article/10.3390/antiox12091788/s1. The document includes additional data regarding color, conversion, TEAC, and DPPH of the model systems as well as HRMS spectra, and tentative assignments of the detected redox clusters. In detail, Figure S1. Browning intensity of (A) the starting solutions of methylglyoxal (MGO) or norfuraneol (NF) with furfural (FF), maltol (MAL), and pyrrole aldehyde (PA) as well as of the individual components at pH 5 measured at 420 nm (red bars: aqueous extracts; brown bars: ethyl acetate extracts). (B) Color of the aqueous solutions (red bars) and their ethyl acetate extracts (brown bars) of the individually treated heterocyclic compounds furfural, maltol, and pyrrole aldehyde after thermal treatment at pH 5 and 130 °C for 120 min. Figure S2. Lightness (L* value) of the (A) aqueous solutions starting solutions at 0 min (light red bars) and 120 min (red bars) as well as of (B) the ethyl acetate extracts. Figure S3. (A) Red/green intensity (a* value) and (B) yellow intensity of the aqueous reaction mixtures (red bars) and their ethyl acetate extracts (brown bars) for the starting solutions of the individual reactants and the binary model mixtures. (C) Red/green intensity (a* value) and (D) yellow intensity (b* value) of individually treated MAL and PA at pH 5 and 130 °C for 120 min. Figure S4. Conversion of FF, PA, and MAL after individual heat treatment at 130 °C and pH for 120 min. Figure S5. Molecular weight distribution as detected by RI (red-dashed line) and at 420 nm (black line) of the reaction mixtures obtained for the individually treated heterocyclic compounds maltol (MAL), furfural (FF), and pyrrole-2-carbaldehyde (PA). As the signal intensities were significantly lower for these individual components, the scaling of the y axis was 50% compared to Figure 4. Figure S6. Antioxidant properties of methylglyoxal (MGO), furfural (FF), and pyrrole-2-carbaldehyde (PA) at the beginning (0 min, light red bars) and after thermal treatment at 130 °C and pH 5 for 120 min (red bars): (A) Aqueous solutions and (B) ethyl acetate extracts. Figure S7. HR-ESI-MS spectra obtained after analysis of the ethyl acetate extracts of MGO/MAL, NF/MAL, MGO/FF, NF/FF, MGO/PA, NF/PA, MGO, and NF. The mass spectra of NF/PA, MGO/MAL, and MGO/FF were modified by removing the signal of the solvent (dimer of ethyl acetate) and re-referencing the intensity of the signals accordingly.
