# Supplementary material for: Colorants and Antioxidants Deriving from Methylglyoxal and Heterocyclic Maillard Reaction Intermediates
Source: Antioxidants (Basel). 2023 Sep 21;12(9):1788. doi: 10.3390/antiox12091788 (PMC10525816; doi:10.3390/antiox12091788)
Supplement: Supplementary file 1 [file antioxidants-12-01788-s001.zip › antioxidants-2627216-supplementary.pdf]

Supplementary Materials

Colorants and Antioxidants Deriving from Methylglyoxal and Heterocyclic MAILLARD Reaction Intermediates

Leon Valentin Bork [1,*], Maximilian Baumann [1], Tobias Stobernack [2], Sascha Rohn [1] and Clemens Kanzler [1,*]

[1] Department of Food Chemistry and Analysis, Institute of Food Technology and Food Chemistry, Technische Universität Berlin, Gustav-Meyer-Allee 25, 13355 Berlin, Germany; m.baumann@tu-berlin.de (M.B.); rohn@tu-berlin.de (S.R.)

[2] Department of Chemical and Product Safety, German Federal Institute for Risk Assessment, Max-Dohrn-Str. 8–10, 10589 Berlin, Germany; tobias.stobernack@bfr.bund.de

* Correspondence: l.bork@tu-berlin.de (L.V.B.); clemens.kanzler@tu-berlin.de (C.K.); Tel.: +49-30-314-72404 (C.K.)

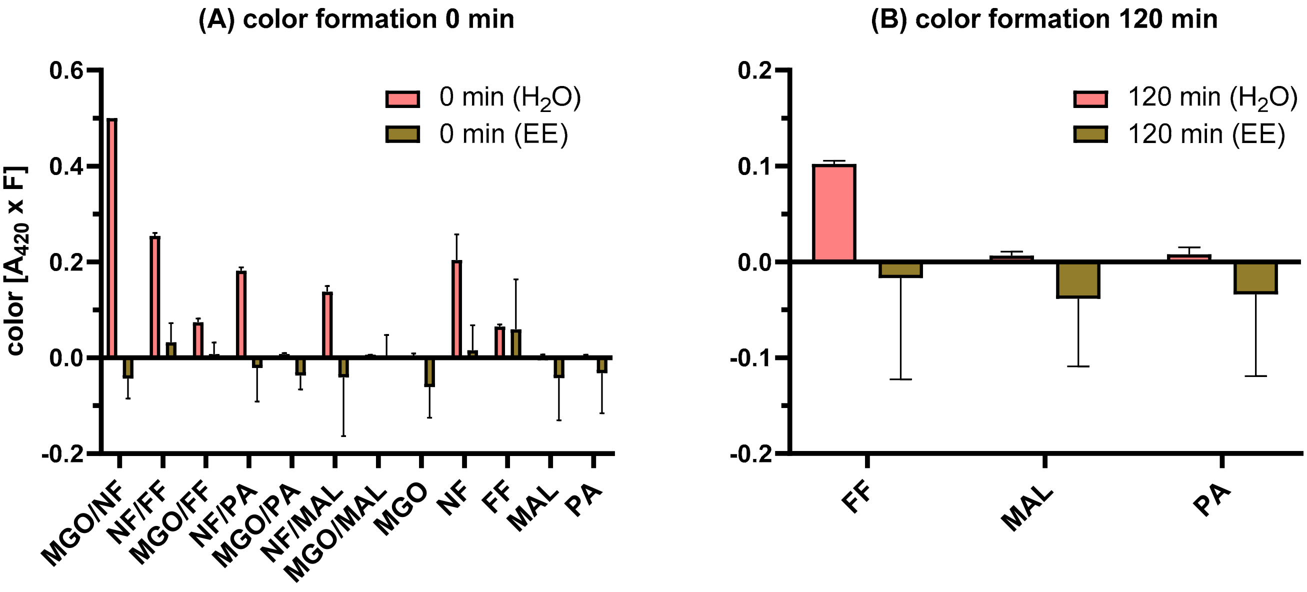

Figure S1. Browning intensity of (A) the starting solutions of methylglyoxal (MGO) or norfuraneol (NF) with furfural (FF), maltol (MAL), and pyrrole aldehyde (PA) as well as of the individual components at pH 5 measured at 420 nm (red bars: aqueous extracts; brown bars: ethyl acetate extracts). (B) Color of the aqueous solutions (red bars) and their ethyl acetate extracts (brown bars) of the individually treated heterocyclic compounds furfural, maltol, and pyrrole aldehyde after thermal treatment at pH 5 and 130 °C for 120 min.

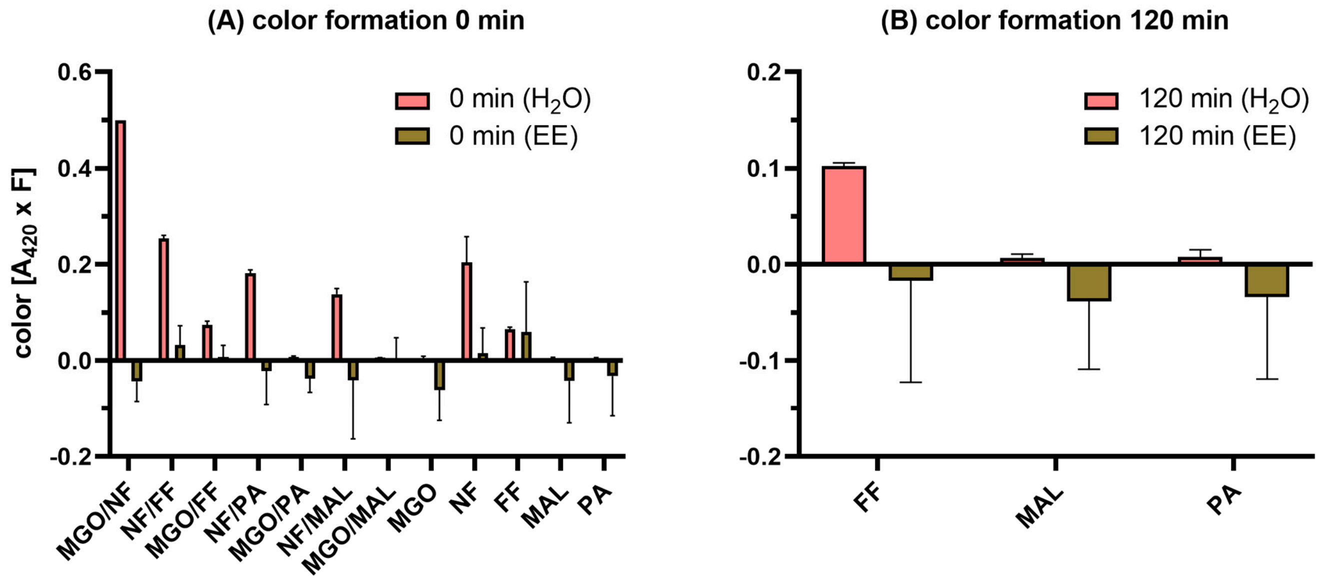

Figure S2. Lightness (L* value) of the (A) aqueous solutions starting solutions at 0 min (light red bars) and 120 min (red bars) as well as of (B) the ethyl acetate extracts.

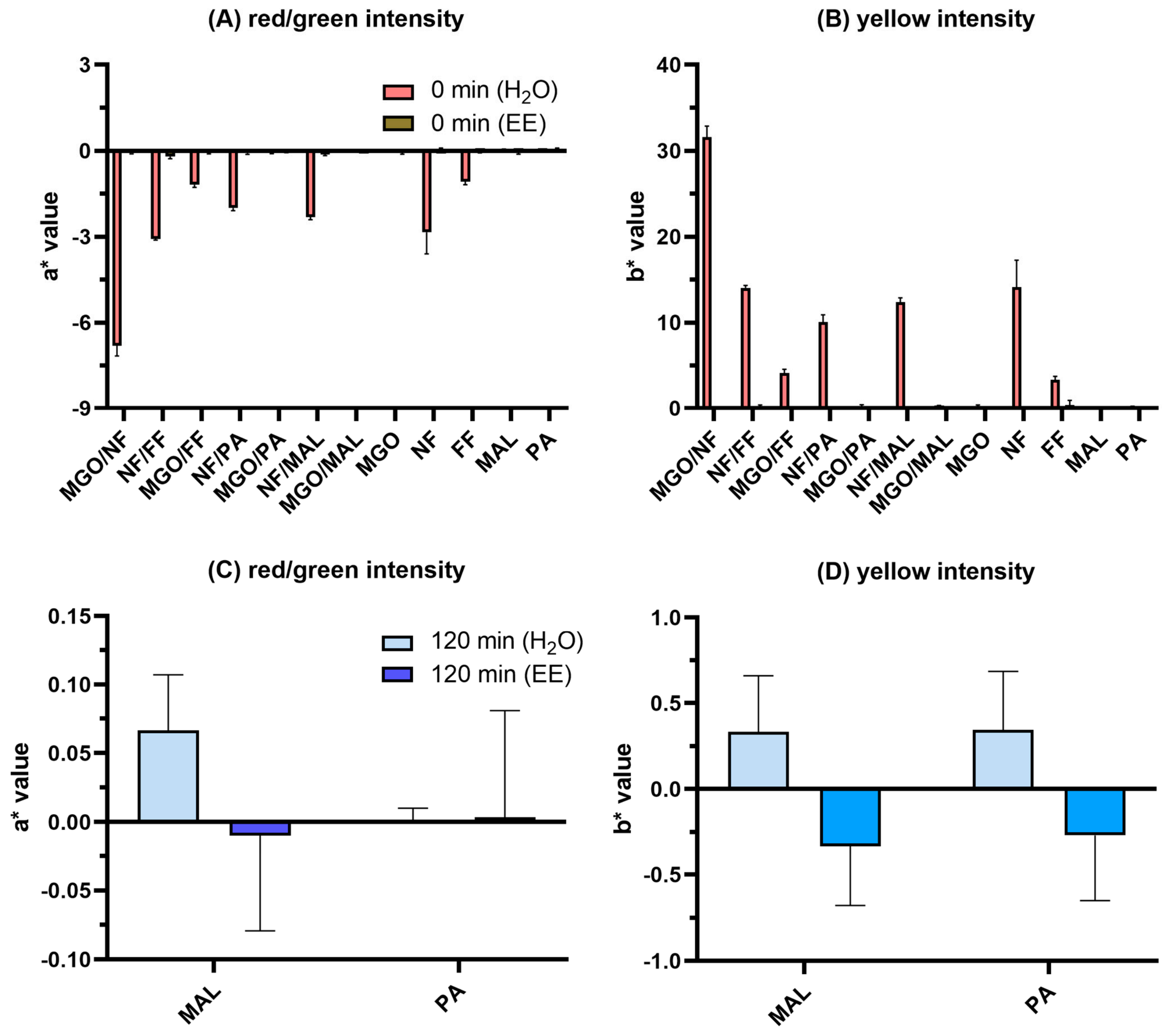

Figure S3. (A) Red/green intensity (a* value) and (B) yellow intensity of the aqueous reaction mixtures (red bars) and their ethyl acetate extracts (brown bars) for the starting solutions of the individual reactants and the binary model mixtures. (C) Red/green intensity (a* value) and (D) yellow intensity (b* value) of Individually treated MAL and PA at pH 5 and 130 °C for 120 min.

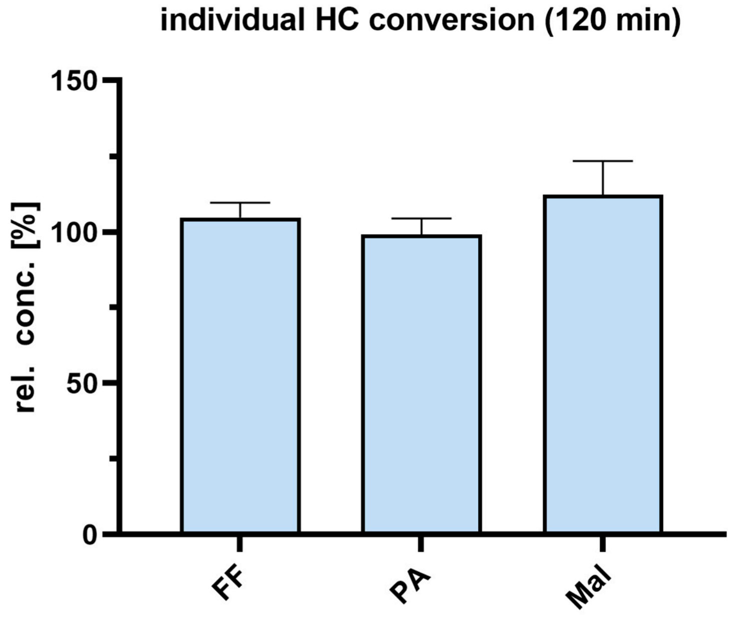

Figure S4. Conversion of FF, PA, and MAL after individual heat treatment at 130 °C and pH for 120 min.

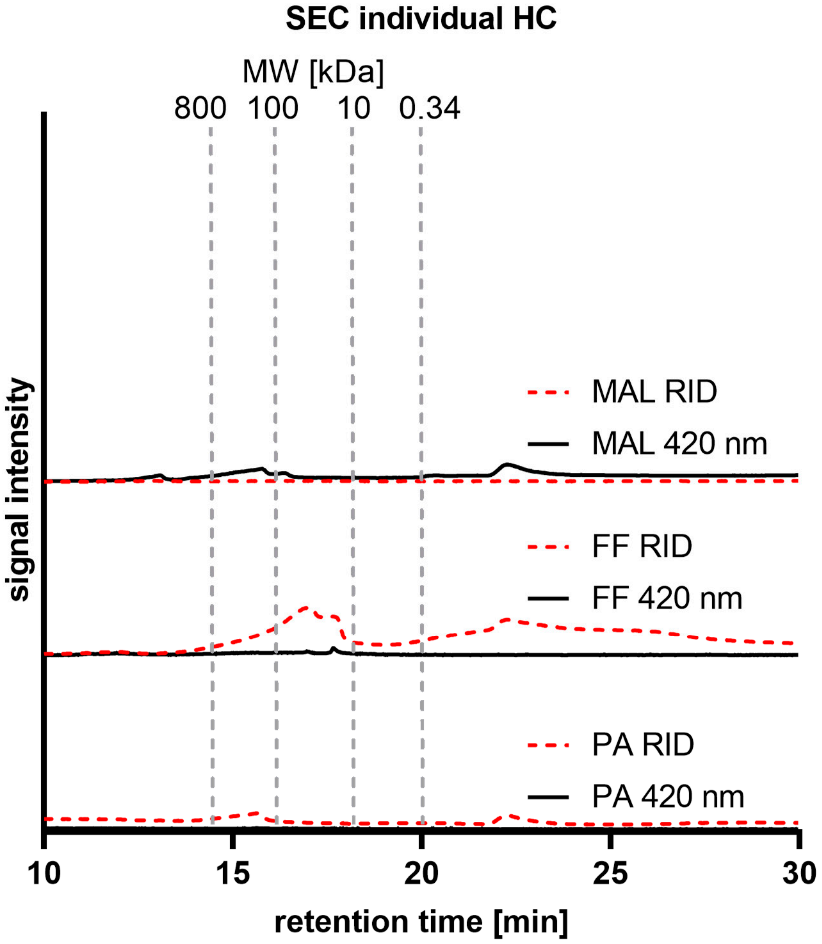

Figure S5. Molecular weight distribution as detected by RI (red-dashed line) and at 420 nm (black line) of the reaction mixtures obtained for the individually treated HCs MAL, FF, and PA. As the signal intensities were significantly lower for these individual components, the scaling of the y axis was 50 % compared to Figure 4.

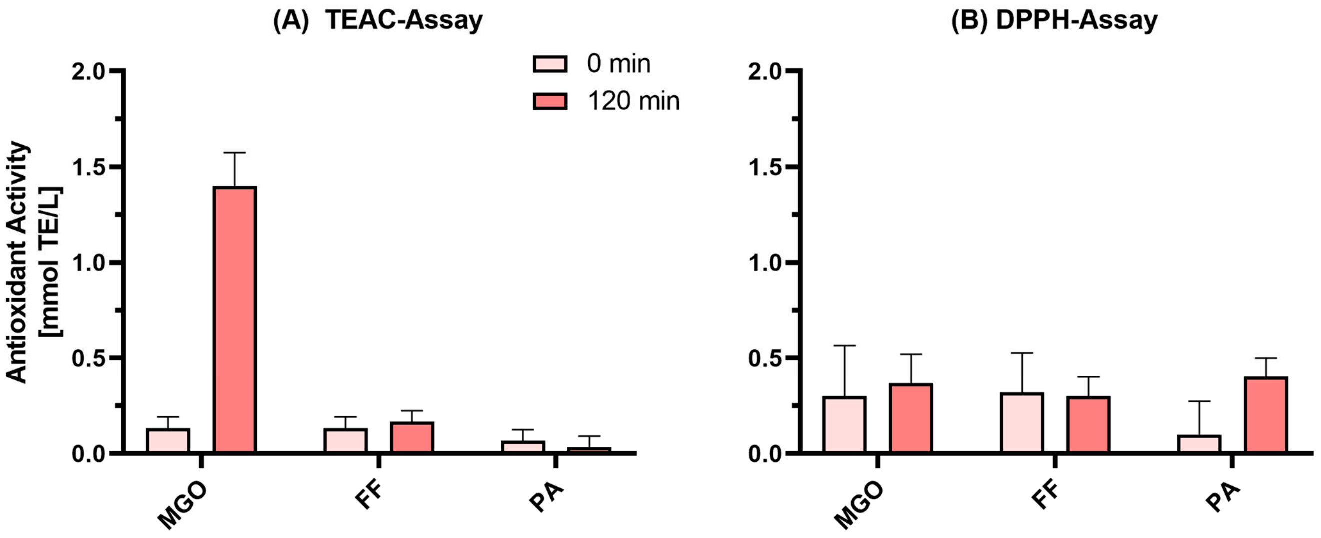

Figure S6. Antioxidant properties of MGO, FF, and PA at the beginning (0 min, light red bars) and after thermal treatment at 130 °C and pH 5 for 120 min (red bars): (A) Aqueous solutions and (B) ethyl acetate extracts.

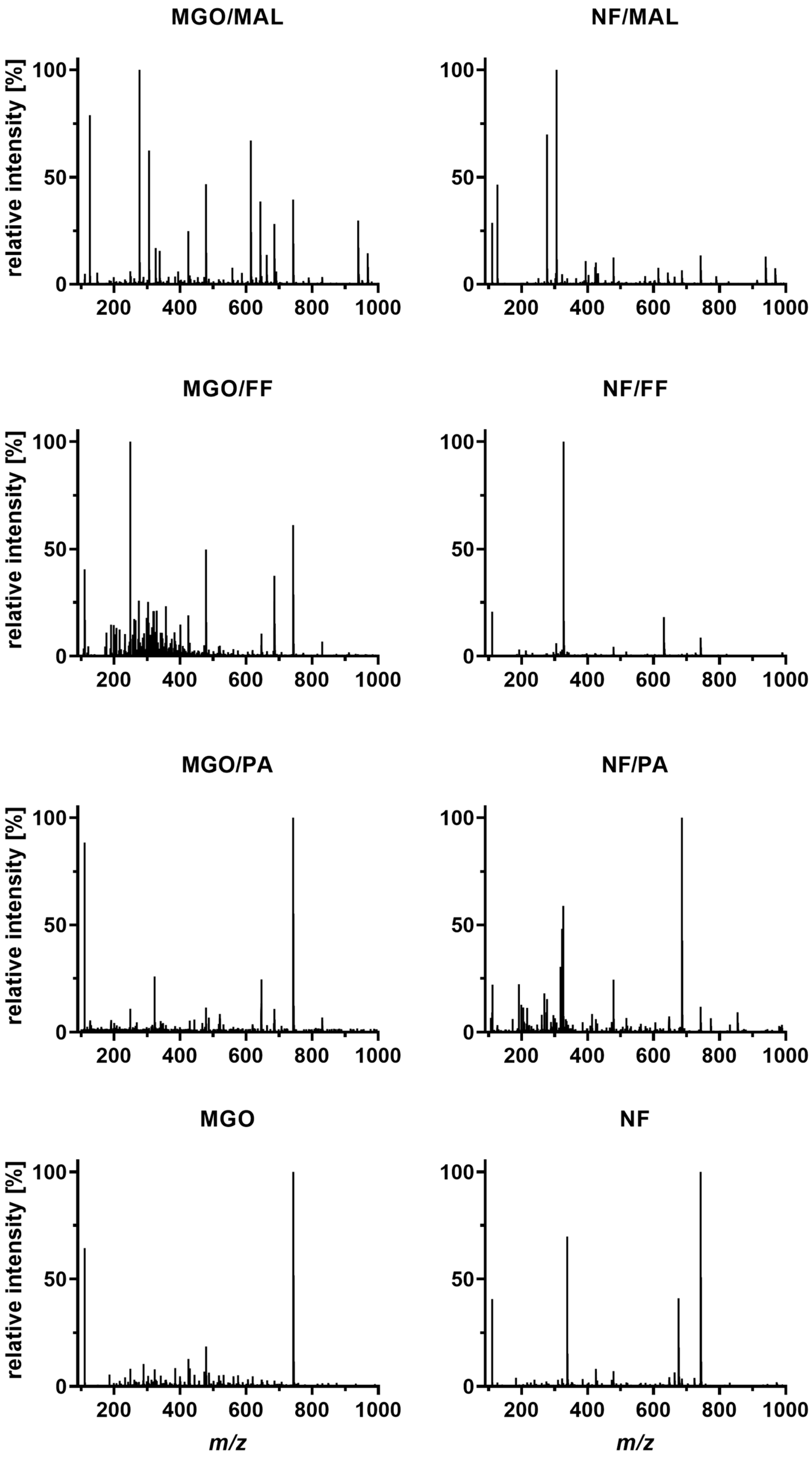

Figure S7. HR-ESI-MS spectra obtained after analysis of the ethyl acetate extracts of MGO/MAL, NF/MAL, MGO/FF, NF/FF, MGO/PA, NF/PA, MGO, and NF. The mass spectra of NF/PA,

MGO/MAL, and MGO/FF were modified by removing the signal of the solvent (dimer of ethyl acetate) and re-referencing the intensity of the signals accordingly.
